# Impact of PCA Pre-Normalization Methods on Ground Reaction Force Estimation Accuracy

**DOI:** 10.3390/s24041137

**Published:** 2024-02-09

**Authors:** Amal Kammoun, Philippe Ravier, Olivier Buttelli

**Affiliations:** 1 PRISME Laboratory, University of Orleans, 12 Rue de Blois, 45100 Orleans, France; amal.kammoun@univ-orleans.fr (A.K.); olivier.buttelli@univ-orleans.fr (O.B.); 2 Emka-Electronique Company, ZA du Patureau de la Grange, 41200 Romorantin-Lanthenay, France; 3 Research Group Sport, Physical Activity, Rehabilitation and Movement for Performance and Health (SAPRèM), University of Orleans, 45100 Orleans, France

**Keywords:** insole measurement, force plate measurement, GRF component estimation, normalization methods, machine learning, PCA pre-normalization

## Abstract

Ground reaction force (GRF) components can be estimated using insole pressure sensors. Principal component analysis in conjunction with machine learning (PCA-ML) methods are widely used for this task. PCA reduces dimensionality and requires pre-normalization. In this paper, we evaluated the impact of twelve pre-normalization methods using three PCA-ML methods on the accuracy of GRF component estimation. Accuracy was assessed using laboratory data from gold-standard force plate measurements. Data were collected from nine subjects during slow- and normal-speed walking activities. We tested the ANN (artificial neural network) and LS (least square) methods while also exploring support vector regression (SVR), a method not previously examined in the literature, to the best of our knowledge. In the context of our work, our results suggest that the same normalization method can produce the worst or the best accuracy results, depending on the ML method. For example, the body weight normalization method yields good results for PCA-ANN but the worst performance for PCA-SVR. For PCA-ANN and PCA-LS, the vector standardization normalization method is recommended. For PCA-SVR, the mean method is recommended. The final message is not to define a normalization method a priori independently of the ML method.

## 1. Introduction

Normalization is a crucial data pre-processing step in machine learning (ML) estimation. Indeed, if measurements have heterogeneous values, quantities with low values are taken into account less than quantities with higher values in the ML estimation procedure. In this way, normalization assigns the same importance to all measurements. The authors in [1] used the robust scaler method in conjunction with an ML method to provide an automated methodology for accurately categorizing various types of defects in industrial IoT ball bearings. The authors in [2] used the min–max normalization method to forecast monthly precipitation. The authors in [3] used the min–max method to estimate position in indoor navigation. The authors in [4] estimated the vertical component of the ground reaction force (GRF) from step sound using body weight normalization. Honert et al. [5] estimated the vertical and anterior–posterior components of the GRF using the Z−score (ZS) normalization method. 

ML methods can also be combined with principal component analysis (PCA), referred to as PCA-ML methods in this paper. Again, it is essential to incorporate a normalization method by centering and normalizing the data points before PCA transformation. PCA transforms a high-dimensional input dataset into a low-dimensional dataset. The motivation behind this transformation is to reduce input data dimensionality, aiming to minimize computational costs in embedded devices. This transformation not only involves reducing input data dimensionality but may also enhance ML estimation performance. The authors in [6] used the min–max method to assess the prediction risk associated with the digital transformation of manufacturing supply chains. The authors in [7] used the ZS method to predict diabetic retinopathy. The authors in [8] used the min–max method to predict power load. Also, ZS normalization is the most commonly suggested method when using PCA, as mentioned in references [9,10,11]. 

This brief state-of-the-art review shows that normalization is required when using ML or PCA-ML methods, whatever the domain of application. However, the authors do not justify why they use or recommend the chosen normalization method. Moreover, normalization methods can be categorized into two classes: statistical approaches and physics approaches. Statistical methods encompass standard techniques, like the min–max and ZS methods. Physics methods rely on the parameters specific to the domain of the database [4,12,13,14]. This is the reason why studying the impact of normalization methods in ML estimation accuracy must be carried out in a specific domain. Our domain is biomechanics. 

In this field, the estimation of GRF components is required in some clinical or biomechanical studies, particularly for the analysis of posture and movement [12]. Instrumented insoles are current low-cost solutions for GRF component estimation. These devices measure pressure through many sensors, from which GRF components can be estimated. Industrial pressure insole systems only evaluate the $F_{z}$ component through a simple linear combination of the pressure sensor values weighted by their individual sensor surface areas. This low-cost approach yields estimation results with limited precision. More sophisticated estimation methods rely on ML principles, which aim to identify the link between insole plantar pressure (PP) data and GRF components in 3D by learning input/output examples. In order to minimize computational costs in instrumented insoles, we focus only on PCA-ML methods to estimate GRF components. 

We hereby propose a detailed state-of-the-art review of normalization methods with the PCA-ML procedure limited to our domain of application. We first introduce the ML methods used. The authors in [12,13,15] employed artificial neural networks (ANNs) in conjunction with PCA to reduce the dimensionality of PP data to estimate GRF components. Rouhani et al. [12] compared the PCA-ANN, PCA-locally linear neuro fuzzy, and PCA–least square (LS) methods for estimating GRF components. Sim et al. [13] compared the three methods presented by Rouhani et al. [12] and also included the PCA-wavelet neural network method in their comparison. The authors of references [12,13] estimated GRF components for walking activities, while Joo et al. [15] focused on estimating GRF components for golf activities. 

Second, we introduce the normalization methods used. These methods may impact the quality of GRF component estimation. While some studies normalized PP data to insole length [12] or to body weight [13] (physics methods), others, like [15], proposed normalization within the range of [–1, 1] (statistical methods). However, none of these authors justified their choice of the normalization method. Additionally, only a few normalization methods have been explored, despite the many existing in the literature.

To the best of our knowledge, no study has evaluated the benefit of PCA methods in combination with normalization approaches. In the present study, we thus propose to assess the impact of twelve different normalization methods from the literature on the accuracy of estimating GRF components. The three components (vertical component ($F_{z}$), anterior–posterior component ($F_{y}$), and medial–lateral component ($F_{x}$)) will be investigated using ANN and LS. This is the first contribution. Also, we will evaluate the performance of the support vector regression (SVR) method, as another comparative method, to estimate GRF components, which has never been tested before in the literature, to the best of our knowledge. This is the second contribution.

To carry out the proposed study, we need to evaluate the estimation accuracy of each PCA-ML method in a supervised context. This is achieved in standardized laboratory conditions by using force plates. The force plates serve as the reference for measured GRF components (ground truth).

**Figure 1:**
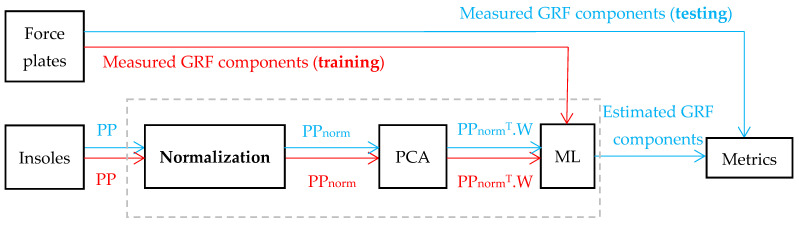
Processing flow chart for estimating GRF components from PP. The PP training set is used for ML modeling by using the corresponding GRF force plate data (in red). The PP testing set is used to evaluate the performance of GRF components by using the corresponding GRF force plate data (in blue). The gray rectangle indicates the classical pre-normalization PCA-ML pipeline.

Figure 1 presents the flow chart for estimating GRF components from PP, where PP represents the input data, PP_norm_ represents the normalized input data, and W is the projection matrix determined by PCA. The estimation accuracy is computed after ML modeling. In the first stage, learning is carried out using the training dataset (red) for modeling. In the second stage, testing is carried out using the test dataset (blue) for computing performance metrics.

Each block of Figure 1 will be detailed in the following section.

## 2. Materials and Methods

### 2.1. Materials and Protocol

**Figure 2:**
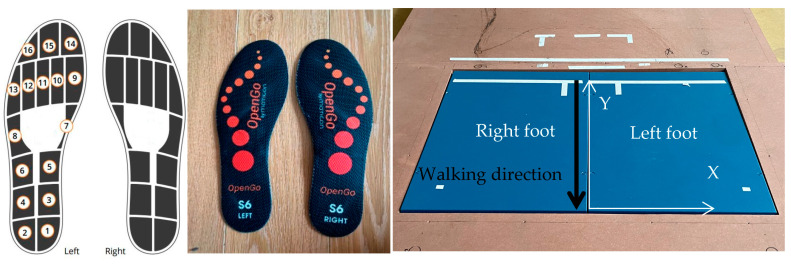
Experimental equipment. **Left**: layout of the pressure sensors of Moticon insoles. **Middle**: Moticon insoles. **Right**: AMTI force plates.

The experimental equipment used in our study included two pressure insole systems (Moticon, ReGo AG Munich, Munich, Germany) equipped with 16 capacitive pressure sensors and 2 force plates (model BMS600900, dimensions of 600 mm × 900 mm; AMTI, Watertown, MA, USA), as shown in Figure 2. 

Nine healthy male subjects (height: 178 ± 4.2 cm; weight: 77 ± 11.1 kg) participated in our study. Before enrollment, participants received detailed information on the study objective and procedure and provide written informed consent, complying with the ethical standards of the Declaration of Helsinki (2013). They wore their own shoes with Moticon insoles, which were of the same size (42 EU). Prior to the experiment, each subject performed three exercises to calibrate the Moticon insoles: a slow walk, standing still, and shifts in body weight. The subjects performed two different tasks on the two force plates to obtain measured GRF component data for both feet (one force plate per foot; Figure 2). These tasks were the following (the reported number indicates the min and max values of steps for each subject):

(1) Normal walking (7–20 steps);
(2) Slow walking (8–22 steps).

The measured GRF component data from the two force plates and the PP data from the Moticon insole systems were sampled at 100 Hz. 

The Fx and Fy components of the force plate frame may not have the same orientation as the foot frame. For the sake of simplification, the Fx and Fy of the force plate frame are considered as the components of the frame of the foot. The foot progression angle (FPA) is used to transform the Fx and Fy components from the force plate frame to the frame of the foot. In our study, the subjects walked in a straight line along the Fy direction (Figure 2), which is the laboratory’s axis of progression (ensured by the protocol that requires the right foot to be placed on one force plate and the left foot on the other force plate). Furthermore, all the subjects participating in this study were healthy (no pathological orientation deviation). In the case of healthy subjects, the study by Caderby et al. [16] showed that estimating the FPA is neither obvious nor standardized and can produce very different FPA values, but the difference is still less than ten degrees. Additionally, we believe that not transforming data with FPA would have little impact on our results. Actually, the FPA value may be tainted by errors, and its addition can impact the quality of GRF component estimation. For these reasons, we did not consider any transformation from the reference frame of the force plates to the frame of the foot.

**Figure 3:**
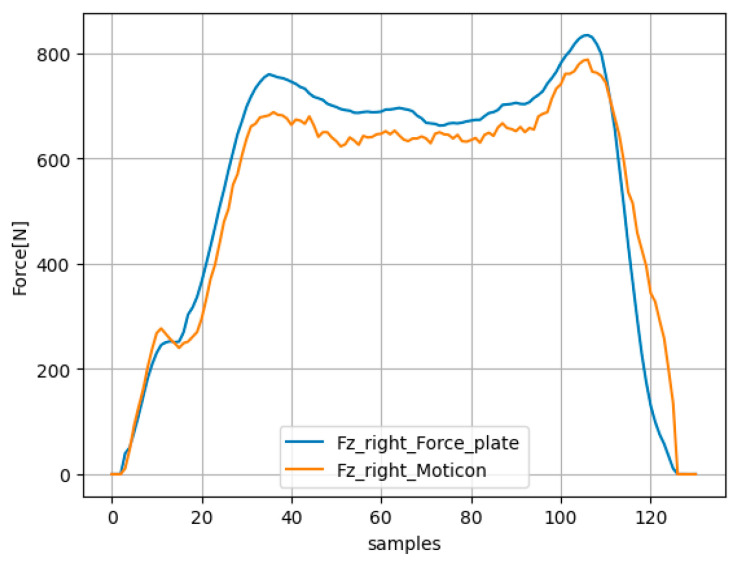
Synchronization by using the time shift with the lowest RMSE between Fz_insole and Fz_force_plate for the right foot during one step.

In the absence of a direct method for digitally or analogically synchronizing force plate data with insole data, we chose a post-processing time-shift synchronization approach for both right and left feet. For this task, we utilized the unique Fz component provided by the Moticon insole system. The Fz component equals the sum of plantar pressure divided by the sensor area over the sensors. We refer to this estimation as Fz_insole. This involves taking the Fz_insole curve and shifting it in relation to the vertical force of the force plate (referred to as Fz_force_plate) curve across a given time range. Subsequently, we compute the root mean square error (RMSE) value and the correlation coefficient (R) as two functions of the time shift. The time-shift value with the lowest RMSE corresponds to the optimal moment for synchronizing the data from the insoles with those from the force plates. A high R value ensures sufficient correlation between the two curves and validates the time-shift value. Figure 3 presents an example of the synchronization by time-shifting between Fz_insole and Fz_force_plate for the right foot during one step.

### 2.2. Normalization Methods

Normalization is an important step for data pre-processing, as it can significantly impact estimation accuracy. Additionally, by ensuring that all variables are equally important and on the same scale, normalization can help speed up the training phase. In our study, we present and apply twelve normalization methods: min–max in the range [0, 1] or [−1, 1], mean, Z-score, robust scaler, vector standardization, maximum linear standardization, decimal scaling, median, and tanh (statistical methods); body weight and length insole (anthropometric/physics methods).

Min–max (MM) [17] is one of the most popular normalizing methods. Given a row of data $X=\left[x_{1},x_{2},\ldots ,x_{n}\right]$, the normalized data using the min–max method in range [0, 1] (MM_[0, 1]_) are given as

(1) $$x_{{\mathrm{norm}}_{i}}=\frac{x_{i}-\min(X)}{\max(X)-\min(X)},\;i=1,\;\ldots ,\;n,$$

where n is the length of the data.

However, this method may not be robust, because it is highly sensitive to outliers [17]. This method can be generalized to adjust the data within the range [a, b] [18]:

(2) $$x_{{\mathrm{norm}}_{i}}=a+\frac{x_{i}-\min(X)}{\max(X)-\min(X)}(b-a).$$

We opted for the max–min method to normalize our data within the range of [−1, 1] (MM_[−1, 1]_). 

The mean method [18] shifts the mean of the data to zero and rescales the dynamic range of the data:

(3) $$x_{{\mathrm{norm}}_{i}}=\frac{x_{i}-\mu (X)}{\max(X)-\min(X)},$$

where μ(X) represents the mean of the data. A drawback of this method is its sensitivity to outliers.

The most classical method is the Z-score (ZS) [17] technique, which centers and reduces data as

(4) $$x_{{\mathrm{norm}}_{i}}=\frac{x_{i}-\mu (X)}{\sigma (X)}.$$

where σ(X) is the standard deviation. The ZS method is less sensitive to outliers than the mean method.

The robust scaler (RS) [18] method is an order statistics-based technique utilized for data that contain outliers. Once normalized, the data exhibit a median of zero:

(5) $$x_{{\mathrm{norm}}_{i}}=\frac{x_{i}-\mathrm{median}(X)}{x_{75}-x_{25}},$$

where $x_{75}$ is the third quartile and $x_{25}$ is the first quartile.

Anysz et al. [19] defined the vector standardization (VS) method as

(6) $$x_{{\mathrm{norm}}_{i}}=\frac{x_{i}}{\sqrt{\sum_{i=1}^{n}x_{i}^{2}}}.$$

They also proposed [19] the maximum linear standardization (MLS) technique. This method involves dividing each sample by the maximum value of the data:

(7) $$x_{{\mathrm{norm}}_{i}}=\frac{x_{i}}{\max(X)}.$$

Decimal scaling (DS) [17] is used particularly when all the different data are distributed on a logarithmic scale. The normalization writes

(8) $$x_{{\mathrm{norm}}_{i}}=\frac{x_{i}}{10^{d}},$$

where d is the number of digits of the maximum absolute value of the data [17]: $d=\log_{10}(\max(\mathrm{abs}(X)).$

The median (Med) normalization method involves dividing each sample by the median of the data [20]:

(9) $$x_{{\mathrm{norm}}_{i}}=\frac{x_{i}}{\mathrm{median}(X)}.$$

The tanh normalization method, as proposed by Hampel et al. [21], can be used to scale data within the range of [0, 1] using the equation provided below:

(10) $$x_{{\mathrm{norm}}_{i}}=0.5\left(\tanh\left(0.01\left(\frac{x_{i}-\mu (X)}{\sigma (X)}\right)\right)+1\right).$$

The last two normalization methods involve some anthropometric measures. The body weight (BW) normalization method divides the data by the body weight (BW) of the subject [14]:

(11) $$x_{{\mathrm{norm}}_{i}}=\frac{x_{i}}{\mathrm{BW}}.$$

The length of the insole (LI) normalization method, proposed by Rouhani et al. [13], divides the data by the length of the insole pressure system:

(12) $$x_{{\mathrm{norm}}_{i}}=\frac{x_{i}}{\mathrm{LI}}.$$

The input (PP) data must be normalized for each pressure sensor when using PCA prior to SVR and LS. In the case of PCA-ANN, the ANN-estimated outputs are normalized values that require denormalization post-processing to retrieve the appropriate magnitude of GRF components. The denormalization method applied to the estimated output is the reverse procedure with respect to the normalization method applied to the input. Note that the authors in [13] propose a specific use of the LI method by denormalizing the estimated output with the BW method.

### 2.3. Principal Component Analysis 

Principal component analysis (PCA) is a technique used to reduce the dimensionality of a dataset (PP data) that contains a large number of dependent variables. The goal is to retain as much of the important information in the dataset as possible [22] by transforming the variables into uncorrelated variables, known as principal components (PCs). Applying PCA to a dataset offers several advantages, such as the following [23]:

(1) It takes less computation time;
(2) Redundant, irrelevant, and noisy data can be removed;
(3) Data quality can be improved;
(4) Some ML methods do not perform well on high-dimensional data. To address this issue and improve accuracy, for example, in ANN, it can be helpful to reduce the dimension of the data.

The steps of PCA [22] begin with the normalization of PP data. Subsequently, we calculate the covariance matrix of the PP data points, from which we calculate the eigenvectors and their corresponding eigenvalues. Finally, we select the k eigenvectors, also called principle components (PCs), that explain the most cumulative variance of the eigenvalues. We use k PCs to create the projection matrix, called W matrix. We transform the data points into a new set with k dimensions using the W matrix. We used PCA to reduce the dimension of the input data from 16 to k PCs, supposing that k was the smallest value for which cumulative variance was higher than 98% [13,16]. 

### 2.4. Machine Learning Methods for GRF Component Estimation

- Artificial Neural Network (ANN)

The artificial neural network (ANN) is a robust algorithm based on the functioning human brain to recognize specific data among a vast number of data and can perform multiple tasks simultaneously. ANN uses a back-propagation network to update the weights between the layers, biases, and activation function parameters to estimate an output closer to the measured output. We conducted a series of experiments on the right foot with varied parameters, following a systematic order. We started with an initial configuration of 2 hidden layers having nodes (128, 256), a batch size of 32, Adamax optimizer, a learning rate of 0.01, activation function set to leaky_relu, and the mean normalization method. We then modified the number of hidden layers and their nodes, which are shown in parentheses, as 1 hidden layer (150); 2 hidden layers (256, 128), (200, 400), (400, 200); 3 hidden layers (600, 400, 200); and 4 hidden layers (800, 600, 400, 200). The optimizer was adjusted by experimenting with Adam, SGD with momentum values of 0.5 and 0.9, and Adamax. We investigated the impact of varying the learning rate, with 0.001, 0.01, 0.05, and 0.1 values. The batch size was systematically altered, exploring values of 1, 16, 32, 64, and 128. Lastly, we explored different activation functions, including sigmoid, relu, tanh, leaky_relu, and wavelet.

**Figure 4:**
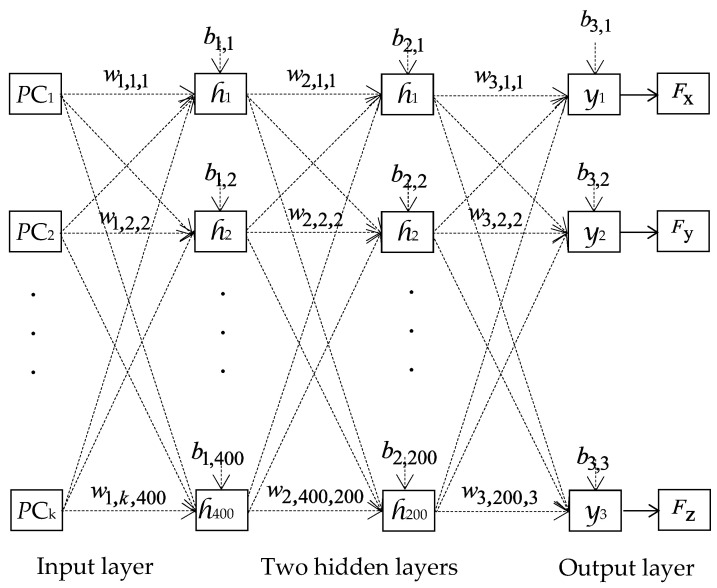
The topology of the ANN for estimating the three GRF components.

The optimal parameters identified from these simulations for ANN modeling, which yielded the highest accuracy of GRF component estimation for the right foot, are as follows: a learning rate of 0.01, a batch size of 32, and the Adamax optimization algorithm. The ANN topology is shown in Figure 4. The left and right insoles are symmetric and for sake of simplification, we make the assumption that the ANN model architectures and parameters for the left foot are identical to those for the right foot.

Figure 4 depicts the network topology used to estimate the GRF components, where input vector $X=\left[PC_{1},PC_{2},\ldots ,PC_{k}\right]$ is followed by two hidden layers, each consisting of 400 and 200 nodes, respectively. The activation function of both hidden layers is represented by *h_i_* , which is chosen to be a relu activation function. The output layer’s activation function is represented by $y_{1}$, $y_{2}$, and $y_{3}$, which stand for the three GRF components.

The outputs of node *j* in the first and second hidden layers are given by the following formula and are denoted as $h_{1}(j)$ and $h_{2}(j)$, respectively:

(13) $$h_{1}(j)=h_{j}(\sum_{i=1}^{k}w_{1,i,j}PC_{i}+b_{1,j}),\;j=1,\;\cdots ,\;n$$

and

(14) $$h_{2}(j)=h_{j}(\sum_{i=1}^{400}w_{2,i,j}PC_{i}+b_{2,j}),\;j=1,\;\ldots ,\;200,$$

where $w_{1,i,j}$, $w_{2,i,j}$, $b_{1,j}$, and $b_{2,j}$ are the weights and biases for both hidden layers, respectively.

The output layer employed the identity activation function, and its calculation is expressed as

(15) $$y(j)=\sum_{i=1}^{200}w_{3,i,j}h_{2}(j)+b_{3,j},\;j=1,\;2,\;3,$$

where $w_{3,i,j}$ is the weight connecting the last hidden layer and the output layer, and $b_{3,j}$ is the bias of the output layer.

The neural network models were implemented using Pytorch (v3.10.7) library and NVIDIA RTX A4500 GPU.

- Least Square (LS) Method

The least square (LS) method is a regression method that allows for finding a linear model that connects the output with the inputs based on knowledge of the experimental data. The fundamental concept behind this approach is centered on minimizing the quadratic criterion between the measured and estimated output quantities, which are related to the chosen mathematical linear model.

- Support Vector Regression (SVR) Method

**Figure 5:**
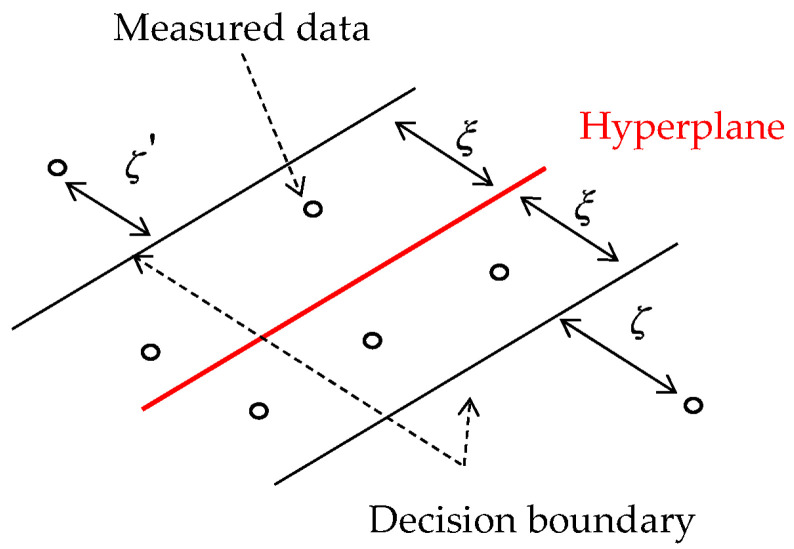
Example of a hyperplane function that maximizes the number of measured data within the decision boundary.

Support vector regression (SVR) is a supervised statistical learning algorithm that is utilized for solving regression problems. For classification tasks, the analogous algorithm SVM (support vector machine) can be employed. The decision boundary is represented by the two black lines in Figure 5, while the red line denotes the hyperplane. 

The distance between the hyperplane and the two decision boundary lines is denoted by ξ, which is a parameter that can be chosen, while the variables $\zeta^{'}$ and ζ indicate the errors between the two decision boundary lines and the measured data.

The objective of this approach is to identify the hyperplane function (which can be a nonlinear function) that maximizes the number of measured data within the decision boundary [24].

We conducted tests with linear and radial basis function (RBF) kernels while varying parameters for the right foot. Specifically, for the linear and RBF kernels, we tested ξ = 15 and 20 with different values of *C*, including 0.1, 1, 10, 50, and 100 (*C* is a regularizing parameter that determines the tolerance for deviations between the kernel and the measured data). Additionally, the supplementary γ parameter with values of 0.1, 1, 10, 50, and 100 was tested for the RBF kernel.

From these simulations, it was found that the RBF kernel model with ξ = 20, *C* = 50, and γ = 10 showed the highest accuracy in estimating GRF components for the right foot. The left and right insoles are symmetric, implying that the SVR model parameters for the left foot are identical to those for the right foot.

The two methods mentioned above (LS and SVR) were implemented using Python (v3.10.7) and an Intel Xeon Gold 5218 R @ 2.10 GHz CPU. 

### 2.5. Machine Learning Modeling 

In order to assess the impact of normalization methods on PCA in conjunction with ANN, LS, and SVR for estimating GRF components, performance indicators were calculated between the estimated (by insole PP data) and measured (by force plate data) GRF components using the whole test datasets of both feet with intrasubject (intras) and intersubject (inters) strategies. The indicators were reported in terms of correlation coefficient (R) and root mean square error (RMSE). 

To construct the ML methods for estimating GRF components, we utilized the datasets of 8 subjects for slow and normal activity for both feet, and 70% of the steps from the datasets for each activity of each subject were used for the training set. The total number of steps for each activity is shown in Table 1. An additional 10% of the steps for each activity were used solely for the validation set of the PCA-ANN model. Our model was evaluated using the intras strategy, which involved testing the model on the test datasets of the same 8 subjects, representing 20% of the steps for each activity. Additionally, we assessed the generalization capacity of our model using the inters strategy, where the whole remaining data of the 9th subject were used for evaluation purposes. Table 1 presents the number of samples and steps for the whole dataset.

For the three ML methods, both intras and inters strategies included the rotation of the training and test datasets to ensure robust results, employing a leave-one-subject-out cross-validation approach. This resulted in the creation of a total of 108 models (9 models for each of the 12 normalization methods) for each foot and each ML method.

### 2.6. Metrics

We used RMSE and *R* coefficient [12,13,15] to evaluate the accuracy of the models for GRF component estimation:

(16) $$\mathrm{RMSE}=\sqrt{\sum_{i=1}^{n}\frac{{\left({\hat{y}}_{i}-y_{i}\right)}^{2}}{n}},[N]$$

and

(17) $$R=\frac{\sum_{i=1}^{n}({\hat{y}}_{i}-\mu (\hat{Y})(y_{i}-\mu (Y))}{\sqrt{\sum_{i=1}^{n}{\left({\hat{y}}_{i}-\mu (\hat{Y})\right)}^{2}\sum_{i=1}^{n}{\left(y_{i}-\mu (Y)\right)}^{2}}}.$$

where n is the number of data points for GRF components; $y_{i}$ is the force plate-measured GRF component ($F_{x}$, $F_{y}$, or $F_{z}$) at time i; ${\hat{y}}_{i}$ is the estimated GRF component; $\mu (\overset{⌢}{Y})$ is the mean of the estimated GRF components; $\overset{⌢}{Y}={\hat{y}}_{1},\ldots ,{\hat{y}}_{n}$ are the estimated GRF components; μ(Y) is the mean of measured GRF components; and $Y=y_{1},\ldots ,y_{n}$ are the measured GRF components.

## 3. Results

### 3.1. Impact of Normalization Methods on PCA for ANN, LS, and SVR for Estimating GRF Components

The nine models from PCA-ANN, LS, and SVR methods were evaluated using RMSE and R metrics for both feet with both strategies. Table 2, Table 3, Table 4 and Table 5 present the average and standard deviation (SD) of these metrics. The values are reported for all normalization methods and the entire test dataset. The best estimation results considering RMSE and R values are highlighted in bold for both **intras** and **inters** strategies. The optimal number of PCs, explaining more than 98% [12,15] of cumulative variance of the PP data, is also presented in these tables. 

After analyzing Table 2 and Table 3, we conducted an analysis to determine the most effective ML method and the corresponding normalization method for estimating GRF components for both feet using the two strategies. For the left foot with the intras strategy, we found that PCA-SVR was the most effective method for estimating $F_{x}$, $F_{y}$ and $F_{z}$ using the mean, MM_[−1, 1]_, and mean normalization methods, respectively. For the inters strategy, PCA-LS was found to be the most effective method for estimating $F_{x}$ and $F_{y}$ using the LI and mean normalization methods, respectively. For the estimation of $F_{z}$, PCA-ANN was the most effective method using the tanh method. Based on the results from Table 4 and Table 5 for the right foot, regarding the intras strategy, PCA-ANN was the most effective method for estimating $F_{x}$, $F_{y}$ and $F_{z}$, using the ZS, tanh and Med and MM_[0, 1]_ normalization methods, respectively. Concerning the inters strategy, for the estimation of $F_{x}$, $F_{y}$ and $F_{z}$, PCA-ANN was the most effective method using the VS and MLS, tanh and VS normalization methods. We can conclude that for the estimation of GRF components, PCA-ANN was the most effective method, followed by PCA-SVR and then PCA-LS in decreasing order of effectiveness.

### 3.2. An Illustration Example of Slow and Normal Walking

**Figure 6:**
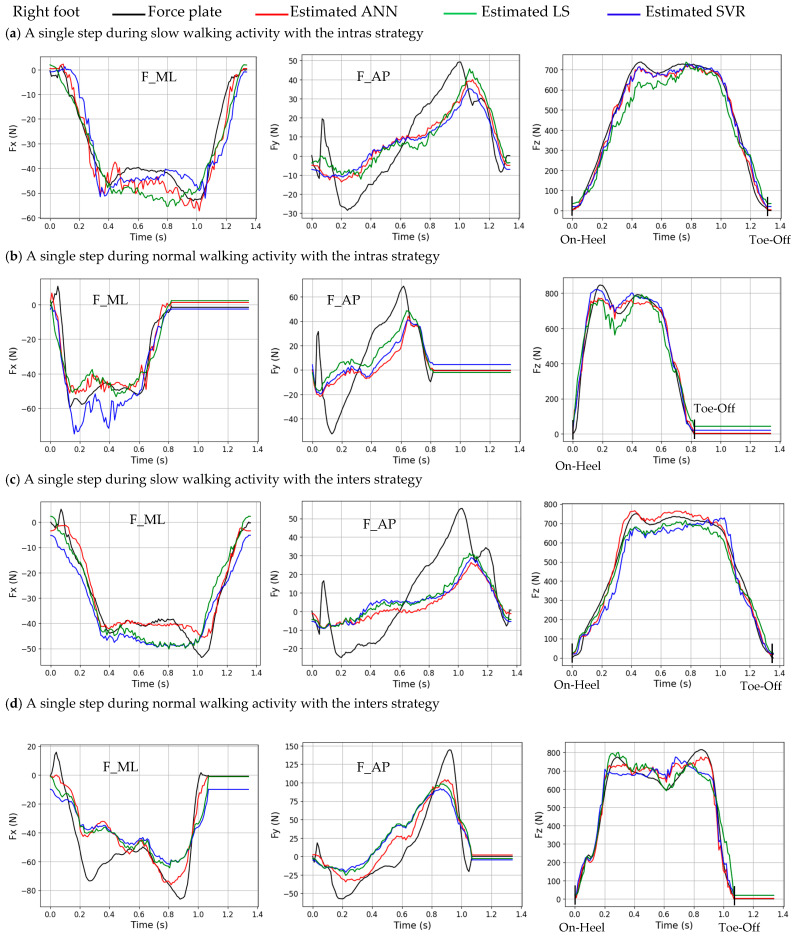
The estimated and measured GRF components for a single step during the “slow walking” and “normal walking” activities for right foot using the intras and inters strategies. The estimated values are represented by the red line for PCA-ANN, the blue line for PCA-SVR, and the green line for PCA-LS. The measured values are represented by the black line.

Figure 6 provides an illustration of the estimated GRF components of a single step of the whole test dataset of the right foot with intras and inters strategies. The normalization technique that produces the most accurate estimation for each ML method in Table 4 and Table 5 is employed to estimate each GRF component (e.g., for the right foot, using the PCA-SVR model, MM_[0, 1]_ is applied to estimate $F_{z}$ with the intras strategy).

Table 6 displays the RMSE and R values for both feet, corresponding to Figure 6, between the estimated and measured GRF components. The RMSE and R values are calculated between the instance of initial contact at the heel (On-Heel) and the instance of foot lift-off (Toe-Off). Based on Table 6, the results of the estimated GRF components by intras and inters strategies during slow walking are more accurate than those during normal walking.

Table 2, Table 3, Table 4, Table 5 and Table 6 indicate that the performance in estimating $F_{x}$ and $F_{y}$ using the optimal normalization method is quite comparable. However, the estimation of the $F_{z}$ component shows a difference, with the PCA-ANN method providing the best outcomes, except in one case. Furthermore, as depicted in Figure 6, in instances where the measured GRF components from the force plate are equal to 0, particularly for the $F_{z}$ component, PCA-LS and PCA-SVR may provide an estimation that differs from 0 due to the constant parameter bias (b). In such cases, PCA-ANN is a better option.

## 4. Discussion

From Table 2, Table 3, Table 4 and Table 5, the *R* value results of our study indicate that the vertical component ($F_{z}$) of the GRF can be estimated more accurately (R_Fz = 0.952–0.979) than the anterior–posterior component (*F_y_*) (R_Fy = 0.606–0.675) and the medial–lateral component (*F_x_*) (R_Fx = 0.634–0.888). These findings are consistent with previous research studies that have also reported similar outcomes [12,13,15] for different estimation methods by using insole measurement. This result holds whatever the strategy and the foot. For instance, Rouhani et al. [12] employed PCA-ANN and PCA-LS, yielding the following *R* values for the three components: R_Fz = 0.991, R_Fy = 0.957–0.960, and R_Fx = 0.933–0.935 with the intras strategy and R_Fz = 0.957–0.967, R_Fy = 0.906–0.908, and R_Fx = 0.764–0.780 with the inters strategy. Sim et al. [13] employed PCA-ANN and PCA-LS, yielding R_Fz = 0.921–0.980, R_Fy = 0.878–0.948, and R_Fx = 0.730–0.912. Joo et al. [15] used PCA-ANN, yielding R_Fz = 0.660–0.950, R_Fy = 0.730–0.830, and R_Fx = 0.650–0.880.

From Table 2, Table 3, Table 4 and Table 5, the metrics show very similar results between right and left feet. This supports the hypothesis stating that both feet can have the same model.

We can note that the optimal results are achieved by PCA-ANN, followed by PCA-SVR and then PCA-LS with 7, 3, and 2 optimal configurations, respectively, among the 12 configurations. The 12 configurations include three components, two feet, and two strategies. The PCA-LS and PCA-SVR both search for the best hyperplane that achieves regression. The latter is linear for LS and nonlinear for SVR (choice of the RBF kernel) which explains why better results were obtained with PCA-SVR than with PCA-LS. Even if SVR provides the best results for some specific configurations (3 out of 12 optimal ones), we recommend the use of ANN, which shows the best results in the majority of configurations (7 out of 12 optimal ones).

From Table 2, Table 3, Table 4, Table 5 and Table 6, it can be inferred that the effectiveness of each normalization technique using PCA varies depending on the ML method (ANN, SVR, or LS) and the strategy (intras or inters) employed for estimating the GRF components of both feet. Contrary to the suggestions of previous studies [9,10,11], our findings suggest that the ZS normalization method may not always be the optimal approach to estimating GRF components when utilizing PCA. For example, using the ZS method to estimate GRF components for both feet with PCA-SVR and both strategies is not recommended because the precision of estimation is low (error values range from 1.2 to 2.7 times greater than the optimal results).

Also, the authors in [12,13] recommended the use of the BW and LI methods to estimate the GRF when employing PCA. However, for PCA-SVR and PCA-LS methods, in the case of $F_{z}$ estimation for both feet with both strategies, these two methods performed poorly, with error values ranging from 1.1 to 4.1 times greater than the optimal results.

In conclusion, for estimating GRF components with both strategies for both feet, we recommend using the VS normalization method with PCA-ANN (error values ranging from 1 to 1.2 times greater than the optimal results) and PCA-LS (error values ranging from 1 to 1.4 times greater than the optimal results). For PCA-SVR, we recommend using the mean method (error values ranging from 1 to 1.2 times greater than the optimal results). Moreover, the same normalization method may produce either poor or excellent accuracy results depending on the ML method employed. It is recommended not to define a normalization method a priori independently of the ML method.

As an example, the BW method can be the least effective for PCA-SVR. For the intras strategy, in the estimation of the $F_{z}$ component of the left foot, this method produced an error value (271.29 N) that is more than three times the optimal error (59.35 N).

In the case where we want to compare or test the performance of the three ML methods by using the same PC data, we recommend using the mean or VS normalization methods for estimating the GRF components with both strategies for both feet. These two methods have shown favorable results with the three ML methods when compared with the optimal results.

PCA can reduce computation time by reducing input dimensionality. However, it requires 16 sensors to reduce the input dimension to 10-6 PCs (Table 2, Table 3, Table 4 and Table 5), depending on the normalization method. This suggests a careful choice of the normalization method for minimizing the number of PCs.

## 5. Conclusions

We conducted a comprehensive study on normalization methods with PCA-ML for GRF estimation, exploring 12 statistical and physical normalization methods instead of the 3 proposed by the authors in [12,13,15]. Moreover, we studied the ZS normalization method, widely used or recommended with PCA [9,10,11] in other application domains. However, the rationale for choosing these methods was not explained. We employed PCA-SVR, a method that, to the best of our knowledge, has never been examined in the literature on GRF component estimation. The PCA-SVR method achieved the second-highest accuracy in estimating GRF components, preceded by PCA-ANN. This paper finally recommends the use of some normalization methods regarding the ML method employed. For PCA-ANN and PCA-LS, the VS normalization method is recommended. For PCA-SVR, the mean method is recommended.

However, our study is limited to normal foot morphology and walking, and further research is needed for other foot characteristics. In other words, for different conditions, the normalization methods with the three ML methods in conjunction with the PCA method need to be reevaluated for estimating GRF components.

We tested only three ML methods. In the future, we plan to explore other ML methods in conjunction with PCA, such as the long short-term memory method. Also, in future work, we aim to select relevant pressure sensors among all the sensors of the insoles using PCA. The selection allows for a reduction in the number of sensors while maintaining high GRF component estimation accuracy.

## Figures and Tables

**Table 1:** The number of samples (steps) of the dataset for the 9 subjects.

Activity	Foot	Dataset
Slow walking	Left	17,732 (128)
	Right	15,008 (112)
Normal walking	Left	12,719 (111)
	Right	11,100 (101)

**Table 2:** Average ± SD of RMSE (R) between the estimated (by insole PP data) and measured (by force plate data) *F_x_* and *F_y_* components for the 9 models using intras and inters strategies with the test dataset of the left foot.

Strategy	Normalization Method	Number of PCs	$F_{x}\left[N\right]$			$F_{y}\left[N\right]$		
			PCA-ANN	PCA-LS	PCA-SVR	PCA-ANN	PCA-LS	PCA-SVR
Intras	MM_[0, 1]_	10-9	13.89 (0.712) ± 0.56 (0.016)	14.97 (0.641) ± 0.67 0.028)	13.83 (0.710) ± 0.51 (0.009)	30.92 (0.647) ± 2.13 (0.032)	33.05 (0.546) ± 1.04 (0.025)	29.85 (0.666) ± 1.80 (0.029)
	MM_[−1, 1]_	10-9	14.12 (0.707) ± 0.87 (0.023)	14.99 (0.639) ± 0.68 (0.028)	14.30 (0.681) ± 0.46 (0.018)	31.48 (0.632) ± 2.51 (0.051)	33.03 (0.547) ± 1.03 (0.024)	**29.04 (0.675) ± 1.13 (0.020)**
	Mean	10-9	14.09 (0.712) ± 1.01 (0.025)	15.12 (0.633) ± 0.78 (0.028)	**13.78 (0.712) ± 0.56 (0.010)**	30.93 (0.643) ± 2.17 (0.038)	33.09 (0.544) ± 1.11 (0.031)	29.24 (0.679) ± 1.70 (0.028)
	ZS	10	13.88 (0.720) ± 1.05 (0.029)	15.11 (0.633) ± 0.77 (0.027)	16.78 (0.549) ± 0.43 (0.020)	30.21 (0.663) ± 1.55 (0.023)	32.60 (0.562) ± 1.07 (0.031)	36.11 (0.452) ± 0.96 (0.027)
	RS	10-9	14.11 (0.706) ± 0.50 (0.015)	15.20 (0.626) ± 0.88 (0.037)	15.36 (0.644) 0.22 (0.019)	30.28 (0.656) ± 1.74 (0.027)	33.10 (0.545) ± 1.32 (0.020)	31.79 (0.607) ± 0.87 (0.024)
	VS	10	14.23 (0.697) ± 0.60 (0.019)	14.92 (0.646) ± 0.69 (0.019)	14.78 (0.656) ± 0.672 (0.021)	31.33 (0.620) ± 1.22 (0.039)	32.85 (0.555) ± 0.84 (0.030)	32.85 (0.551) ± 0.84 (0.026)
	MLS	10-9	14.11 (0.709) ± 0.70 (0.027)	14.97 (0.641) ± 0.67 (0.028)	13.83 (0.710) ± 0.51 (0.009)	30.95 (0.644) ± 2.50 (0.042)	33.05 (0.546) ± 1.04 (0.025)	29.85 (0.666) ± 1.80 (0.029)
	DS	6	14.82 (0.658) ± 0.83 (0.031)	16.03 (0.559) ± 0.66 (0.072)	15.18 (0.624) ± 0.63 (0.047)	31.92 (0.599) ± 1.68 (0.041)	33.04 (0.547) ± 1.00 (0.023)	31.30 (0.607) ± 1.28 (0.024)
	Med	9-8	15.40 (0.655) ± 0.99 (0.033)	14.94 (0.640) ± 0.73 (0.029)	17.57 (0.494) ± 0.49 (0.009)	31.94 (0.613) ± 1.04 (0.035)	33.16 (0.545) ± 1.33 (0.031)	37.44 (0.362) ± 0.89 (0.035)
	Tanh	10	15.20 (0.621) ± 0.72 (0.030)	15.17 (0.630) ± 0.69 (0.021)	15.19 (0.633) ± 0.77 (0.026)	32.85 (0.550) ± 0.80 (0.049)	33.10 (0.542) ± 1.09 (0.049)	33.19 (0.535) ± 0.93 (0.048)
	BW	9	14.80 (0.699) ± 1.01 (0.018)	15.35 (0.648) ± 0.45 (0.024)	19.29 (0.257) ± 0.63 (0.014)	31.55 (0.627) ± 1.59 (0.044)	33.66 (0.534) ± 1.25 (0.030)	39.30 (0.006) ± 1.15 (0.001)
	LI	9-8	14.47 (0.686) ± 0.54 (0.016)	15.26 (0.621) ± 0.76 (0.029)	19.13 (0.282) ± 0.61 (0.024)	30.88 (0.647) ± 1.44 (0.035)	33.52 (0.530) ± 0.86 (0.023)	39.30 (0.013) ± 1.15 (0.008)
Inters	MM_[0, 1]_	10-9	17.48 (0.528) ± 3.92 (0.191)	15.10 (0.610) ± 3.42 (0.138)	17.68 (0.473) ± 2.97 (0.139)	38.09 (0.538) ± 8.74 (0.123)	32.62 (0.638) ± 6.62 (0.088)	37.94 (0.527) ± 7.06 (0.097)
	MM_[−1,1]_	10-9	17.96 (0.528) ± 4.58 (0.203)	15.16 (0.613) ± 3.38 (0.140)	16.33 (0.546)± 4.40 (0.117)	38.53 (0.574) ± 9.56 (0.091)	32.61 (0.639) ± 6.63 (0.088)	35.34 (0.513) ± 9.77 (0.114)
	Mean	10-9	18.85 (0.525) ± 4.64 (0.191)	14.87 (0.623) ± 3.86 (0.143)	17.14 (0.502)± 3.50 (0.159)	38.95 (0.529) ± 13.6 (0.201)	**31.43 (0.652) ± 6.29 (0.086)**	37.75 (0.528) ± 7.83 (0.084)
	ZS	10	17.32 (0.526) ± 4.89 (0.205)	15.12 (0.587) ± 2.96 (0.139)	17.52 (0.514)± 4.23 (0.087)	41.60 (0.528) ± 12.2 (0.134)	32.32 (0.619) ± 6.93 (0.091)	39.58 (0.175) ± 11.1 (0.071)
	RS	10-9	18.01 (0.532) ± 4.93 (0.227)	14.88 (0.593) ± 3.64 (0.195)	16.94 (0.547) ± 4.46 (0.102)	36.43 (0.585) ± 9.60 (0.077)	32.28 (0.634) ± 7.27 (0.082)	37.70 (0.370) ± 11.3 (0.085)
	VS	10	14.66 (0.602) ± 3.32 (0.155)	14.56 (0.620) ± 2.83 (0.136)	14.94 (0.623) ± 3.60 (0.149)	32.62 (0.649) ± 6.72 (0.053)	32.07 (0.637) ± 6.71 (0.085)	31.60 (0.636) ± 6.77 (0.085)
	MLS	10-9	18.49 (0.525) ± 5.60 (0.201)	15.10 (0.610) ± 3.42 (0.138)	17.68 (0.473) ± 2.97 (0.139)	38.89 (0.528) ± 11.7 (0.183)	32.62 (0.638) ± 6.62 (0.088)	37.94 (0.527) ± 7.06 (0.097)
	DS	6	18.23 (0.521) ± 5.52 (0.198)	17.26 (0.524) ± 4.54 (0.150)	16.88 (0.532) ± 4.62 (0.159)	33.89 (0.615) ± 7.11 (0.071)	32.62 (0.625) ± 6.48 (0.094)	32.52 (0.631) ± 6.69 (0.065)
	Med	9-8	16.51 (0.567) ± 4.88 (0.152)	14.68 (0.601) ± 3.14 (0.148)	17.93 (0.486) ± 4.24 (0.113)	35.35 (0.606) ± 7.85 (0.060)	31.99 (0.634) ± 7.07 (0.094)	39.68 (0.131) ± 11.3 (0.087)
	Tanh	10	15.07 (0.616) ± 3.58 (0.138)	14.78 (0.595) ± 2.88 (0.136)	15.14 (0.605) ± 3.26 (0.140)	31.81 (0.638) ± 6.99 (0.071)	32.29 (0.620) ± 6.92 (0.092)	31.50 (0.638) ± 7.09 (0.080)
	BW	9	17.52 (0.567) ± 4.15 (0.106)	16.00 (0.598) ± 3.85 (0.139)	19.14 (0.286) ± 3.73 (0.081)	41.60 (0.513) ± 10.0 (0.138)	32.97 (0.624) ± 7.14 (0.074)	40.06 (0.006) ± 11.0 (0.003)
	LI	9-8	17.26 (0.530) ± 3.43 (0.150)	**14.49 (0.634) ± 3.11 (0.109)**	19.04 (0.310) ± 3.77 (0.082)	42.90 (0.463) ± 12.3 (0.233)	33.03 (0.610) ± 8.52 (0.097)	40.06 (0.018) ± 11.0 (0.017)

**Table 3:** Average ± SD of RMSE (R) between the estimated (by insole PP data) and measured (by force plate data) $F_{z}$ component for the 9 models using intras and inters strategies with the test dataset of the left foot.

Strategy	Normalization Method	Number of PCs	$F_{z}\left[N\right]$		
			PCA-ANN	PCA-LS	PCA-SVR
Intras	MM_[0, 1]_	10-9	63.13 (0.977) ± 4.15 (0.003)	83.36 (0.957) ± 12.67 (0.013)	59.99 (0.979) ± 4.39 (0.003)
	MM_[−1, 1]_	10-9	64.70 (0.976) ± 4.63 (0.003)	84.66 (0.956) ± 12.27 (0.013)	73.40 (0.969) ± 1.62 (0.002)
	Mean	10-9	62.84 (0.978) ± 3.95 (0.002)	82.50 (0.959) ± 8.73 (0.008)	**59.35 (0.979) ± 3.16 (0.002)**
	ZS	10	63.38 (0.977) ± 5.15 (0.004)	77.53 (0.964) ± 7.22 (0.007)	160.81 (0.837) ± 6.03 (0.012)
	RS	10-9	71.06 (0.971) ± 6.78 (0.005)	96.11 (0.941) ± 22.12 (0.028)	108.83 (0.931) ± 5.96 (0.009)
	VS	10	72.93 (0.970) ± 5.55 (0.004)	83.13 (0.958) ± 6.43 (0.008)	87.15 (0.956) ± 11.69 (0.012)
	MLS	10-9	66.40 (0.975) ± 5.77 (0.004)	83.36 (0.957) ± 12.67 (0.013)	59.99 (0.979) ± 4.39 (0.003)
	DS	6	108.91 (0.941) ± 24.48 (0.020)	123.45 (0.886) ± 42.53 (0.092)	104.16 (0.924) ± 33.72 (0.059)
	Med	9-8	112.89 (0.928) ± 13.25 (0.017)	101.34 (0.936) ± 15.50 (0.022)	185.00 (0.776) ± 7.14 (0.016)
	Tanh	10	81.53 (0.959) ± 10.25 (0.010)	79.49 (0.962) ± 7.37 (0.007)	85.32 (0.96) ± 14.24 (0.011)
	BW	9	61.84 (0.978) ± 4.67 (0.003)	109.14 (0.945) ± 7.68 (0.006)	271.29 (0.442) ± 6.35 (0.009)
	LI	9-8	72.49 (0.969) ± 5.71 (0.005)	84.87 (0.956) ± 12.36 (0.013)	264.95 (0.478) ± 6.24 (0.010)
Inters	MM_[0, 1]_	10-9	107.47 (0.944) ± 25.07 (0.020)	106.96 (0.940) ± 31.80 (0.030)	112.41 (0.942) ± 42.21 (0.021)
	MM_[−1, 1]_	10-9	110.61 (0.936) ± 25.71 (0.016)	106.95 (0.941) ± 31.81 (0.030)	118.08 (0.934) ± 50.36 (0.030)
	Mean	10-9	102.51 (0.942) ± 17.42 (0.025)	98.15 (0.947) ± 25.42 (0.025)	106.51 (0.946) ± 40.92 (0.022)
	ZS	10	106.30 (0.933) ± 29.23 (0.029)	98.63 (0.949) ± 24.00 (0.018)	183.11 (0.808) ± 36.42 (0.046)
	RS	10-9	120.47 (0.912) ± 38.21 (0.086)	117.99 (0.919) ± 54.84 (0.085)	146.61 (0.888) ± 45.29 (0.044)
	VS	10	91.65 (0.949) ± 24.34 (0.013)	101.65 (0.949) ± 36.60 (0.027)	98.20 (0.951) ± 31.74 (0.030)
	MLS	10-9	114.30 (0.938) ± 32.34 (0.017)	106.96 (0.94) ± 31.80 (0.030)	112.41 (0.942) ± 42.21 (0.021)
	DS	6	149.84 (0.904) ± 54.20 (0.047)	145.49 (0.896) ± 73.81 (0.090)	120.89 (0.921) ± 59.96 (0.052)
	Med	9-8	127.73 (0.905) ± 40.76 (0.035)	95.79 (0.945) ± 20.06 (0.022)	197.21 (0.766) ± 3360 (0.073)
	Tanh	10	**90.14 (0.952) ± 19.02 (0.019)**	96.70 (0.952) ± 26.18 (0.020)	97.39 (0.951) ± 16.28 (0.023)
	BW	9	95.87 (0.949) ± 27.95 (0.026)	115.54 (0.946) ± 35.99 (0.024)	269.09 (0.471) ± 25.96 (0.041)
	LI	9-8	147.79 (0.923) ± 72.68 (0.032)	108.84 (0.953) ± 35.87 (0.019)	264.68 (0.497) ± 25.53 (0.041)

**Table 4:** Average ± SD of RMSE (R) between the estimated (by insole PP data) and measured (by force plate data) $F_{x}$ and $F_{y}$ components for the 9 models using intras and inters strategies with the test dataset of the right foot.

Strategy	Normalization Method	Number of PCs	$F_{x}\left[N\right]$			$F_{y}\left[N\right]$		
			PCA-ANN	PCA-LS	PCA-SVR	PCA-ANN	PCA-LS	PCA-SVR
Intras	MM_[0, 1]_	10-9	11.35 (0.887) ± 0.56 (0.009)	13.86 (0.823) ± 0.58 (0.013)	12.51 (0.857) ± 0.72 (0.011)	33.47 (0.624) ± 1.75 (0.033)	31.67 (0.624) ± 1.37 (0.026)	31.64 (0.646) ± 1.50 (0.025)
	MM_[−1, 1]_	10-9	11.73 (0.881) ± 0.50 (0.008)	13.67 (0.829) ± 0.73 (0.011)	13.82 (0.825) ± 0.825 (0.010)	32.00 (0.641) ± 1.20 (0.024)	31.73 (0.622) ± 1.40 (0.026)	32.66 (0.610) ± 1.79 (0.033)
	Mean	10-9	11.40 (0.885) ± 0.58 (0.009)	13.86 (0.823) ± 0.59 (0.013)	12.49 (0.858) ± 0.73 (0.012)	33.34 (0.621) ± 1.51 (0.031)	31.67 (0.624) ± 1.376 (0.026)	31.55 (0.649) ± 1.68 (0.028)
	ZS	10	**11.28 (0.888) ± 0.65 (0.013)**	13.66 (0.829) ± 0.75 (0.027)	17.84 (0.699) ± 0.84 (0.016)	32.38 (0.648) ± 1.75 (0.048)	31.67 (0.625) ± 1.50 (0.015)	37.65 (0.425) ± 1.47 (0.039)
	RS	10-9	11.68 (0.880) ± 0.60 (0.010)	13.38 (0.837) ± 0.64 (0.012)	16.34 (0.753) ± 0.79 (0.019)	33.12 (0.630) ± 2.06 (0.040)	31.76 (0.621) ± 1.27 (0.015)	35.93 (0.502) ± 1.78 (0.046)
	VS	10	11.75 (0.876) ± 0.55 (0.012)	13.34 (0.838) ± 0.36 (0.011)	13.50 (0.835) ± 0.44 (0.011)	31.34 (0.653) ± 1.44 (0.029)	31.65 (0.624) ± 1.04 (0.023)	31.26 (0.636) ± 1.03 (0.021)
	MLS	10-9	11.51 (0.883) ± 0.48 (0.008)	13.86 (0.823) ± 0.58 (0.013)	12.50 (0.857) ± 0.72 (0.011)	32.32 (0.636) ± 1.33 (0.032)	31.67 (0.624) ± 1.37 (0.026)	31.64 (0.646) ± 1.50 (0.025)
	DS	6	12.79 (0.853) ± 0.55 (0.014)	14.51 (0.804) ± 1.18 (0.027)	13.66 (0.830) ± 0.91 (0.014)	32.16 (0.624) ± 1.90 (0.030)	32.12 (0.612) ± 1.28 (0.016)	31.26 (0.637) ± 1.49 (0.013)
	Med	9-8	14.88 (0.811) ± 1.64 (0.032)	14.33 (0.809) ± 0.92 (0.028)	19.37 (0.615) ± 0.75 (0.020)	**31.63 (0.665) ± 2.13 (0.030)**	31.56 (0.627) ± 1.04 (0.021)	39.39 (0.292) ± 1.86 (0.061)
	Tanh	10	13.36 (0.836) ± 0.63 (0.020)	13.64 (0.829) ± 0.82 (0.028)	14.26 (0.818) ± 0.84 (0.027)	**30.80 (0.649) ± 1.25 (0.014)**	31.46 (0.631) ± 1.32 (0.013)	31.17 (0.644) ± 1.24 (0.012)
	BW	9	12.55 (0.862) ± 0.80 (0.018)	14.70 (0.799) ± 0.97 (0.025)	22.91 (0.390) ± 0.88 (0.005)	33.33 (0.613) ± 2.12 (0.057)	31.94 (0.615) ± 1.35 (0.024)	40.53 (0.002) ± 1.76 (0.004)
	LI	9-8	11.53 (0.883) ± 0.52 (0.013)	14.03 (0.819) ± 0.72 (0.015)	22.59 (0.431) ± 0.86 (0.009)	32.78 (0.621) ± 1.96 (0.049)	32.01 (0.611) ± 1.23 (0.035)	40.52 (0.032) ± 1.76 (0.011)
Inters	MM_[0, 1]_	10-9	16.79 (0.777) ± 5.51 (0.064)	14.43 (0.799) ± 2.72 (0.065)	15.19 (0.786) ± 2.53 (0.082)	47.92 (0.434) ± 23.01 (0.180)	34.01 (0.594) ± 8.89 (0.099)	39.48 (0.429)± 9.67 (0.127)
	MM_[−1, 1]_	10-9	16.18 (0.770) ± 3.45 (0.071)	14.57 (0.796) ± 2.85 (0.067)	16.75 (0.765) ± 3.34 (0.078)	44.49 (0.450) ± 19.78 (0.161)	34.22 (0.589) ± 8.81 (0.094)	37.96 (0.388) ± 11.41 (0.090)
	Mean	10-9	17.62 (0.776) ± 8.06 (0.078)	14.54 (0.797) ± 2.87 (0.068)	15.15 (0.787) ± 2.48 (0.080)	41.33 (0.486) ± 14.35 (0.147)	34.04 (0.593) ± 8.93 (0.098)	39.12 (0.438) ± 9.08 (0.130)
	ZS	10	15.38 (0.779) ± 3.35 (0.080)	15.29 (0.788) ± 3.18 (0.067)	19.42 (0.654) ± 3.50 (0.073)	42.61 (0.481) ± 12.71 (0.154)	34.30 (0.584) ± 9.75 (0.095)	39.86 (0.140) ± 12.56 (0.053)
	RS	10-9	16.12 (0.739) ± 3.81 (0.110)	15.35 (0.772) ± 3.37 (0.084)	18.56 (0.695) ± 3.37 (0.070)	47.21 (0.482) ± 15.43 (0.124)	33.29 (0.599) ± 10.02 (0.081)	40.00 (0.225) ± 12.17 (0.052)
	VS	10	**14.14 (0.798) ± 3.25 (0.089)**	14.25 (0.796) ± 2.35 (0.078)	14.21 (0.798) ± 2.26 (0.082)	39.03 (0.515) ± 12.48 (0.169)	33.70 (0.587) ± 9.40 (0.085)	33.21 (0.601) ± 9.35 (0.086)
	MLS	10-9	**14.53 (0.810) ± 2.70 (0.067)**	14.42 (0.799) ± 2.72 (0.065)	15.19 (0.786) ± 2.53 (0.082)	42.61 (0.520) ± 18.24 (0.138)	34.01 (0.594) ± 8.89 (0.099)	39.48 (0.429) ± 9.67 (0.127)
	DS	6	15.57 (0.744) ± 2.98 (0.122)	15.34 (0.765)± 2.76 (0.100)	14.72 (0.774) ± 2.91 (0.094)	37.21 (0.523) ± 9.74 (0.122)	33.20 (0.592) ± 9.26 (0.097)	33.14 (0.591) ± 9.35 (0.108)
	Med	9-8	17.82 (0.740) ± 4.69 (0.097)	15.051 (0.79) ± 2.17 (0.076)	20.07 (0.602) ± 3.48 (0.072)	43.86 (0.518) ± 22.05 (0.154)	33.21 (0.610) ± 9.82 (0.081)	39.62 (0.081) ± 12.72 (0.045)
	Tanh	10	14.50 (0.802) ± 2.61 (0.064)	15.19 (0.788) ± 3.17 (0.065)	15.54 (0.792) ± 2.65 (0.061)	**33.07 (0.606) ± 9.43 (0.099)**	34.38 (0.585) ± 9.40 (0.094)	33.57 (0.597) ± 9.42 (0.093)
	BW	9	16.96 (0.757) ± 4.76 (0.079)	16.00 (0.797) ± 3.19 (0.066)	22.66 (0.401) ± 3.96 (0.043)	44.02 (0.435) ± 15.34 (0.200)	33.73 (0.587) ± 8.64 (0.114)	39.56 (0.010) ± 12.99 (0.015)
	LI	9-8	16.09 (0.770) ± 4.25 (0.099)	14.99 (0.780)± 2.16 (0.088)	22.46 (0.429) ± 3.96 (0.047)	41.44 (0.469) ± 9.86 (0.079)	33.92 (0.576) ± 10.47 (0.109)	39.55 (0.038) ± 12.99 (0.019)

**Table 5:** Average ± SD of RMSE (R) between the estimated (by insole PP data) and measured (by force plate data) $F_{z}$ component for the 9 models using intras and inters strategies with the test dataset of the right foot.

Strategy	Normalization Method	Number of PCs	$F_{z}\left[N\right]$		
			PCA-ANN	PCA-LS	PCA-SVR
Intras	MM_[0, 1]_	10-9	**65.68 (0.976) ± 3.36 (0.003)**	93.66 (0.948) ± 4.43 (0.006)	67.34 (0.974) ± 2.04 (0.002)
	MM_[-1,1]_	10-9	66.15 (0.975) ± 2.39 (0.002)	91.02 (0.951) ± 7.18 (0.007)	76.20 (0.967) ± 3.06 (0.002)
	Mean	10-9	66.37 (0.976) ± 3.60 (0.003)	93.76 (0.948) ± 4.611 (0.006)	67.40 (0.974) ± 1.92 (0.002)
	ZS	10	67.35 (0.974) ± 2.90 (0.003)	102.31 (0.936) ± 18.19 (0.024)	163.65 (0.834) ± 7.49 (0.014)
	RS	10-9	71.24 (0.971) ± 3.38 (0.003)	102.71 (0.935) ± 19.12 (0.024)	127.77 (0.904) ± 6.03 (0.009)
	VS	10	78.23 (0.966) ± 3.67 (0.003)	92.26 (0.949) ± 10.17 (0.012)	99.00 (0.942) ± 13.17 (0.015)
	MLS	10-9	71.57 (0.972) ± 5.05 (0.004)	93.66 (0.948) ± 4.43 (0.006)	67.36 (0.974) ± 2.04 (0.002)
	DS	6	92.22 (0.952) ± 13.58 (0.011)	118.30 (0.912) ± 24.58 (0.037)	99.08 (0.940) ± 17.06 (0.019)
	Med	9-8	116.90 (0.929) ± 11.55 (0.011)	105.90 (0.931) ± 19.61 (0.028)	204.91 (0.725) ± 4.80 (0.011)
	Tanh	10	98.21 (0.942) ± 12.17 (0.015)	102.53 (0.936) ± 15.51 (0.021)	119.46 (0.919) ± 19.15 (0.026)
	BW	9	67.36 (0.974) ± 3.99 (0.003)	114.76 (0.93) ± 16.609 (0.021)	270.02 (0.476) ± 5.38 (0.007)
	LI	9-8	74.35 (0.969) ± 2.65 (0.001)	97.69 (0.943) ± 11.852 (0.013)	262.60 (0.513) ± 5.67 (0.011)
Inters	MM_[0, 1]_	10-9	93.94 (0.949) ± 24.97 (0.027)	102.28 (0.952) ± 24.87 (0.024)	101.99 (0.948) ± 38.26 (0.019)
	MM_[−1, 1]_	10-9	92.96 (0.949) ± 24.41 (0.035)	110.95 (0.943) ± 36.74 (0.052)	123.07 (0.928) ± 46.17 (0.029)
	Mean	10-9	93.88 (0.949) ± 22.20 (0.030)	110.01 (0.941) ± 36.95 (0.052)	102.77 (0.946) ± 38.62 (0.022)
	ZS	10	91.65 (0.949) ± 22.68 (0.027)	97.48 (0.944) ± 26.36 (0.020)	185.59 (0.798) ± 38.15 (0.043)
	RS	10-9	104.01 (0.936) ± 30.59 (0.04)	123.92 (0.914) ± 47.41 (0.067)	164.64 (0.852) ± 40.75 (0.034)
	VS	10	**89.82 (0.952) ± 30.34 (0.024)**	97.09 (0.944)± 33.64 (0.036)	106.37 (0.932) ± 48.24 (0.059)
	MLS	10-9	95.37 (0.953) ± 25.75 (0.032)	102.28 (0.952) ± 24.87 (0.024)	101.99 (0.948) ± 38.26 (0.019)
	DS	6	134.25 (0.888) ± 50.72 (0.082)	140.46 (0.879) ± 49.44 (0.094)	111.04 (0.912) ± 28.92 (0.060)
	Med	9-8	151.27 (0.888) ± 44.57 (0.044)	109.51 (0.925) ± 38.04 (0.069)	209.15 (0.73) ± 33.75 (0.051)
	Tanh	10	103.94 (0.942) ± 38.79 (0.032)	98.25 (0.942) ± 28.44 (0.026)	116.92 (0.931) ± 35.51 (0.039)
	BW	9	93.64 (0.949) ± 20.28 (0.026)	128.14 (0.954) ± 50.48 (0.023)	264.46 (0.501) ± 30.29 (0.038)
	LI	9-8	147.34 (0.922) ± 53.39 (0.053)	101.09 (0.940) ± 28.65 (0.036)	259.22 (0.529) ± 30.39 (0.040)

**Table 6:** RMSE (R) between the estimated (by insole PP data) and measured (by force plate data) GRF components for slow and normal walking using intras and inters strategies for the right foot, corresponding to Figure 6. The most accurate normalization method for each ML method in Table 4 and Table 5 is used to estimate each GRF component.

Strategy	Activity	Method	$F_{x}\left[N\right]$	$F_{y}\left[N\right]$	$F_{z}\left[N\right]$
Intras	Slow	PCA-ANN	**5.15 (0.975)**	**11.17 (0.709)**	**26.60 (0.996)**
		PCA-SVR	7.40 (0.912)	12.43 (0.681)	29.03 (0.995)
		PCA-LS	6.92 (0.948)	12.63 (0.669)	68.49 (0.980)
	Normal	PCA-ANN	**6.88 (0.946)**	24.84 (0.425)	48.95 (0.988)
		PCA-SVR	10.53 (0.941)	**21.12 (0.577)**	**45.33 (0.989)**
		PCA-LS	8.91 (0.911)	23.61 (0.484)	75.18 (0.973)
Inters	Slow	PCA-ANN	**3.42 (0.981)**	17.33 (0.701)	**33.68 (0.993)**
		PCA-SVR	6.84 (0.941)	16.07 (0.673)	60.69 (0.981)
		PCA-LS	6.39 (0.930)	**15.15 (0.736)**	44.42 (0.994)
	Normal	PCA-ANN	**15.29 (0.879)**	**18.61 (0.889)**	**44.42 (0.984)**
		PCA-SVR	18.87 (0.808)	28.49 (0.691)	64.23 (0.965)
		PCA-LS	17.54 (0.837)	26.71 (0.727)	66.23 (0.965)

## Data Availability

Data are unavailable for public sharing due to confidentiality.
